# Developing a Program Theory on Ventilator Weaning in Adult Intensive Care: Protocol for a Multimethods Study

**DOI:** 10.2196/83342

**Published:** 2026-01-30

**Authors:** Fritz Sterr, Lydia Bauernfeind, Christian Rester, Sabine Metzing, Rebecca Palm

**Affiliations:** 1 School of Nursing Sciences Faculty of Health Witten/Herdecke University Witten Germany; 2 Faculty of Applied Healthcare Sciences Deggendorf Institute of Technology Deggendorf Germany; 3 Department of Health Services Research School VI Medicine and Health Sciences Carl von Ossietzky Universität Oldenburg Oldenburg Germany

**Keywords:** artificial respiration, critical care, intensive care, mechanical ventilation, program theory, study protocol, ventilator weaning

## Abstract

**Background:**

Worldwide, mechanical ventilation and ventilator weaning have been widely researched. Nevertheless, rates of weaning failure remain high. According to the Medical Research Council framework, ventilator weaning is a complex intervention. While there are various guidelines on this, there is no abstract theoretical understanding that organizes the interventions, outcomes, and their contexts.

**Objective:**

This study aims to explore the interconnectedness of interventions, outcomes, and context in ventilator weaning of adult intensive care patients.

**Methods:**

Using the approach of Funnell and Rogers, we develop a program theory for ventilator weaning in a multimethod study comprising 2 main steps. First, 3 literature reviews on interventions and outcomes, predictors of weaning failure, and patients’ experiences were triangulated with stakeholder conversations. Using abduction, we then developed an initial program theory. Second, the initial theory will be revised in an iterative process. To this end, semistructured group discussions and workshops will be conducted, followed by a deductive thematic analysis and adaptation of our theory. This process will be repeated until stakeholder statements and data analyses are congruent with the program theory.

**Results:**

The initial program theory developed in step 1 is presented in this protocol and serves as the basis for review and refinement in step 2. The results of this iterative process and the final program theory are expected in 2026.

**Conclusions:**

Following the Medical Research Council framework, a program theory on ventilator weaning will be developed in this study. This may enable a differentiated understanding of ventilator weaning and more sustainable and comprehensive research. The program theory emphasizes the interdisciplinary nature of ventilator weaning and supports health care professionals in combining interventions appropriately and evaluating relevant outcomes.

**Trial Registration:**

Open Science Framework YGJ3T; https://doi.org/10.17605/OSF.IO/YGJ3T

**International Registered Report Identifier (IRRID):**

DERR1-10.2196/83342

## Introduction

### Background

Worldwide, an increasing number of patients receive mechanical ventilation (MV) in an intensive care unit (ICU) [1-6]. The indication for MV is usually due to acute respiratory insufficiency [7-11]. Other reasons are acute shock, coma, or planned surgical procedures [11,12]. While MV is an important and life-saving intervention in these situations, it also leads to several negative consequences.

It can cause endotracheal tube–related complications (eg, displacement, laryngeal injury, and tracheomalacia) [11], physical consequences (eg, cardiovascular and respiratory collapse, inflammation, ventilator-induced lung injury, peripheral organ injury, and impaired cognition) [11,13-15], and psychological consequences (eg, anxiety, stress, indifference, depression, and posttraumatic stress disorder) [16,17].

It is of central importance to initiate ventilator weaning at an early stage. To this end, a broad body of evidence is already available to guide this process. Various risk factors [18-20], interventions [21-24], and the patient’s experience of weaning [25-27] have been empirically investigated, and the nursing diagnosis “dysfunctional adult ventilatory weaning response” has been developed [28,29]. These findings are summarized in various international guidelines [8,30,31].

### Problem Definition

The Medical Research Council (MRC) framework defines a complex intervention according to several criteria. These include “the number of components, the range of behaviors targeted; expertise and skills required by those delivering and receiving the intervention; the number of groups, settings, or levels targeted; or the permitted level of flexibility” [32]. Following this understanding and the information in the Background section, ventilator weaning is to be understood as a complex intervention.

For a complex intervention to be developed and effective in the long term, it requires a theory-based understanding of which interventions interact, which outcomes they affect, and which context they depend on [32]. Despite the fact that MV and ventilator weaning have been investigated extensively, treatment in ICUs has not yet reached its optimum. To date, less than two-thirds of patients can be successfully weaned from the respirator [12,33,34]. In the Weaning According to a New Definition (WIND) study, 24.3% never entered the weaning process [33], and in the Worldwide Assessment of Separation of Patients From Ventilatory Assistance (WEAN SAFE) study, 28.3% died under MV [12]. While these findings can be explained by various reasons, more than 50% of the patients previously declared unweanable could still be weaned after their ICU stay [35]. This is a problem in that the longer durations of MV are associated with a higher mortality rate [33].

In addition, most studies only examine singular aspects of the weaning process. While this is of central importance for drawing clear conclusions about the effects of interventions and consequently making recommendations for or against them, it does not represent the real embedding of these interventions in complex intensive care [36]. As described by the MRC framework, it is essential to understand the interplay of interventions, outcomes, and their context. Therefore, reducing ventilator weaning to isolated aspects is a cause for concern. It is not a simple but a multimodal and multilayered process in which a large number of factors must be considered [20]. Thus, further research into weaning methods is repeatedly demanded, for example, in the context of patients with neurological conditions [37,38].

Two major research gaps emerge. On the one hand, most research studies only answer the question of “whether,” but not “how and why.” Nevertheless, these questions need to be answered as required by the MRC framework [32]. The limited perspective makes it impossible to understand the real influences and causal relationships of weaning interventions, outcomes, and their context. This finding is reinforced by the fragmentary nature of the studies conducted to date; there is a lack of evidence linking the interventions. The required opening of the black box does not take place at this point [39-42].

By contrast, there is a substantial deficit in the theoretical understanding of ventilator weaning. While no grand theory could be identified, only 1 middle-range theory on the nursing diagnosis “dysfunctional adult ventilatory weaning response” was found [29]. However, this theory only covers “weaning failure” and its antecedents and clinical consequents. In addition, only practical guidelines for clinicians appear at this stage that lack abstraction and theoretical conceptualization. A theory covering the entire weaning process in the ICU is still missing. This results in the versatile use of the term “ventilator weaning,” which has changed in its classification, definition, and understanding over the years [33,43] and is the basis of an inconsistent, incoherent research logic [44]. This is another reason why an international consensus in MV research and the necessary theory development have recently been requested [45].

### Aim and Research Questions

To conceptualize, develop, and organize ventilator weaning as a complex program in the long term, this study is conducted in line with the recommendations of the MRC framework. The central aim of this study is to explore the interconnectedness of individual interventions in ventilator weaning with relevant outcomes and their context.

Specifically, ventilator weaning as a program, the interconnection of interventions, and the associated factors should be described comprehensively. This is followed by an elaboration of the central mechanisms in ventilator weaning. Particularly, the focus is on the contribution of the identified interventions to changing the individual endpoints, which should also be formulated. On the basis of these aims, three research questions were formulated: (1) How is ventilator weaning composed in adult intensive care patients? (2) What results can be achieved through ventilator weaning in adult intensive care patients? and (3) How can the mechanisms in the process of ventilator weaning be theoretically summarized and comprehensively depicted?

## Methods

### Overview

To answer the research questions, a program theory is to be developed in line with the method of Funnell and Rogers [46]. The development of a program theory is the method of choice in this project, as it is explicitly proposed as a core element for complex interventions by the MRC framework [32] and can find answers to the central questions of this study. This study is oriented toward the SPIRIT (Standard Protocol Items: Recommendations for Interventional Trials) guideline [47,48] and the relevant aspects herein, as there are currently no reporting guidelines for qualitative studies or program theory development. The study was registered in the Open Science Framework (YGJ3T) on May 14, 2025.

### Philosophical Background of Program Theories

The ontological and epistemological classification of program theories is a complex process that requires various considerations. A current and differentiated discussion assigns program theories to ontological realism [49]. This assumes that real structures and processes exist and work on their own, regardless of what we know or experience. In addition, a clear distinction is to be made between the transitive domain (including social structures and cause-and-outcome relationships that can change over time) and the intransitive domain (understanding and interpreting reality at a deeper level).

Furthermore, this study [49] allocates program theories to epistemological relativism. This means that knowledge and truth are relative to specific cultural or conceptual frameworks or settings. They note that researchers need to consider “the multi-causal nature of social structures and grasp their complexity through causal mechanisms leading to outcomes” [49].

In this regard, program theories are mainly understood as technological theories that are assigned to evaluation research and find themselves in theory-driven or theory-based approaches [46,50,51].

### Methodological Framework

#### Overview

This study builds on the methodological guidance of Funnell and Rogers [46]. To develop a program theory, they recommend an iterative process in which various data sources are included and triangulated with each other. In detail, they refer to the combination of three approaches: (1) deductive development (eg, existing literature and reviews), (2) inductive development (eg, group discussions and interviews), and (3) articulating program stakeholders’ mental models (eg, stakeholder conversations and workshops) [46].

Following these recommendations, the approach of this study is structured along two central steps: (1) the development of an initial program theory and (2) the review and revision of the program theory. The first step is already complete, and the results obtained will be presented in this study together with the planned revision of the theory.

**Figure 1:**
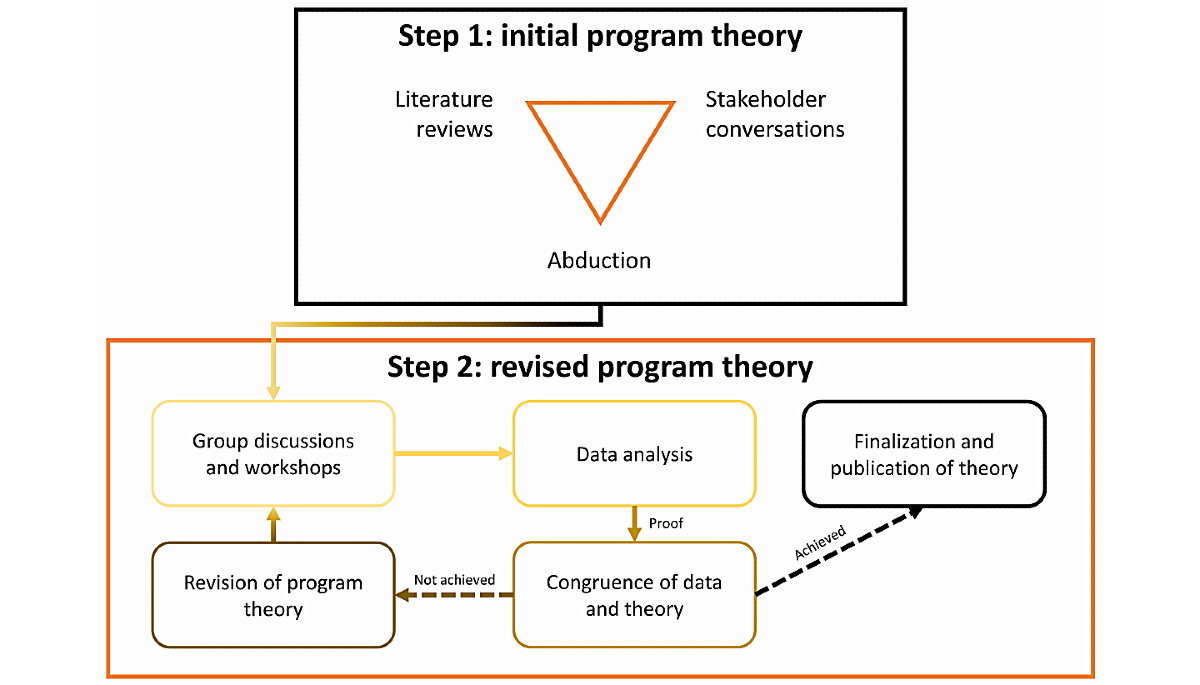
Methodological approach to developing the program theory.

Figure 1 illustrates the overarching methodological approach of the study. It depicts the 2 phases and the interconnections of the various steps in this approach. These are described in detail subsequently.

#### Step 1: Developing the Initial Program Theory

According to the underlying methodology [46], a program theory consists of 2 core components: the “theory of action” (ToA; related to research questions 1 and 3) and the “theory of change” (ToC; related to research questions 2 and 3). In addition, there is usually a logic model that narratively and/or graphically depicts how the ToA affects the ToC and induces the changes.

In detail, the ToC “refers to the central mechanism by which change comes about for individuals, groups, and communities” [46]. It consists of a comprehensive situation analysis, the focus and scope of the program, and the outcomes chain as “the centerpiece of the program theory” [46]. The ToA “explains how programs or other interventions are constructed to activate their theory of change” [46]. It contains desired attributes of outcomes, program features, and external factors affecting the outcomes and the activities of the program and external factors [46].

To develop a program theory, Funnell and Rogers [46] recommend starting with a comprehensive situation analysis. Researchers should identify what is already known about the program, what evidence base exists, and what still needs to be researched, and, above all, they are asked for “the nature and extent of the main problem that the program addresses” [46]. This step is followed by focusing and shaping the planned program and theory, setting clear boundaries. Subsequently, key outcomes and their attributes are derived, the interventions are identified, and, finally, the interrelationships in this program are reflected upon. The ToA and ToC can then be developed from this. In this study, the development of the initial program theory comprised the triangulation of data from (1) literature analysis, (2) stakeholder conversations, and (3) an abductive process.

As MV is widely researched, 3 systematic reviews were conducted in the literature phase. In a scoping review, interventions associated with ventilator weaning and related patient outcomes were identified [52]. In the next step, predictors of weaning failure were compiled in a systematic evidence map as observational criteria for the program theory [53]. Finally, the perspective of those affected was also examined. For this purpose, a meta-synthesis was carried out to gain an in-depth understanding of patients’ experiences under MV [54].

Following the completion of the initial literature phase, 13 stakeholder conversations were held with various health care professionals (HCPs) between January 2025 and April 2025. These conversations were conducted using a semistructured interview guide and documented with field notes. According to the recommendations of Funnell and Rogers [46], these conversations were intended for stakeholders to articulate their initial mental models. In addition to a nurse and a physiotherapist, 2 physicians (an anesthesiologist and an intensivist), 2 critical care advanced practice nurses, 3 senior nursing researchers, and a paramedic were interviewed. The interview guide (Multimedia Appendix 1) included questions on the causality and indication of MV, its aim and associated problems, the initiation of weaning, relevant outcomes and their attributes in the process, key interventions, weaning failure, and influencing factors from outside. The triangulation of data from the literature phase and the stakeholder conversations was carried out in MAXQDA (version 24; VERBI Software GmbH), followed by a deductive analysis according to the key terminology of Funnell and Rogers [46], and resulted in an initial program theory.

In addition, the initial theory was complemented and expanded by the first author through an abductive process. This is defined as a subconscious process that occurs involuntarily and spontaneously as the only creative step in the entire research process. The abduction itself cannot be justified; only its results can be justified [55]. Therefore, we have identified additional connections in the intervention and outcome chain that are integrated into the initial program theory. It is important to note that this process alone may not result in accurate statements [56]. Therefore, results from the abductive process are embedded in a multimethod approach, and statements derived are critically examined in the revision of our theory.

To discuss the overall comprehensibility and consistency of the initial theory development, 2 social scientists and a sociologist were consulted, who are familiar with theory development but have no connection to intensive care medicine.

#### Step 2: Revision of the Program Theory

To evaluate and revise the initial theory, an iterative process is recommended that draws, in particular, on stakeholders and practitioners [46]. We have designed a cyclical process for this purpose, which is illustrated in Figure 1.

We will hold semistructured group discussions [57,58] and workshops [46] with various HCPs in 4-hour sessions every 3 to 4 weeks. These will focus on outcomes, interventions, the context, and their interrelationships. The interview guides for the group discussions and the workshops are provided in Multimedia Appendix 1. Each session will be audio recorded, transcribed verbatim, and then undergo a reflective thematic analysis based on a deductive approach [59-61].

Subsequently, the current program theory and the collected data are assessed for congruence. If no congruence is achieved, the program theory will be adapted to the data collected and reviewed again in group discussions and workshops with other study participants. This iterative process will be repeated until congruence of data and program theory has been achieved. If there is congruence, the program theory will then be finalized and published.

### Study Setting

The study will be conducted at the Deggendorf Institute of Technology in Bavaria, Germany. Participants will meet with colleagues from other institutions independently of their own institution. This will create a space of openness and mutual inspiration.

### Eligibility Criteria

People who have completed training in a health care profession (eg, medicine, nursing, physiotherapy, occupational therapy, speech therapy, or respiratory therapy), are aged 18 years or older, and have 12 months of experience in treating ICU patients receiving MV can participate in the study. In addition, they must participate voluntarily in the study and be able to give informed consent.

### Sample Size

To include different professions and perspectives and allow each participant to speak sufficiently, the sample size for each session is of crucial importance. Therefore, we recruit 6 to 10 people per group discussion so that not too many people are involved at the same time, and interaction between participants, follow-up questions, and an in-depth exploration of the subject matter are still possible.

### Recruitment

Purposive sampling was used in this study for initial stakeholder conversations in step 1. To this end, 1 or 2 representatives from each relevant health care profession were requested for a conversation by the first author. Participants had to be experts in their field and hold at least a master’s degree and have 5 years of professional experience. As this was an informal conversation and only field notes were taken, but no personal data were recorded, the participants had to declare their voluntary participation verbally; however, they did not have to sign an informed consent.

In step 2, purposive sampling will be used for the revision of the program theory. To this end, potential participants are informed about the study via various channels. They can then voluntarily contact the study coordinator if they wish to participate. As a first step, the professional networks of the study authors are used, and the information regarding the study is disseminated there. When potential participants respond, they are assessed by the researchers for their suitability. Following the ontological realism on which program theory is based, we aim to represent the actual staffing composition in ICUs as realistically as possible (unlike in step 1). Therefore, in the assessment, we look for a broad distribution of skills and degrees, tasks and roles, as well as professional experience. If participation is too low, the relevant professional and scientific societies in Germany will be contacted in the second step, and information regarding this study will be spread within them.

### Researcher Reflexivity

Reflecting on the role and previous knowledge of the researchers themselves is of great importance in empirical studies [62]. All 5 researchers involved in this study are registered nurses and have at least several years of professional experience in the ICU. They all have actively cared for MV patients themselves and accompanied them through the process of ventilator weaning. Currently, only 1 author (LB) still works part time in the ICU. All 5 authors work at different universities—3 (CR, RP, and S Metzing) as professors and 2 (LB and FS) as research associates. One author is a specialist in health services research and the development of program theories (RP), and another is a specialist in qualitative research methods (S Metzing). One author deals primarily with professional development and nursing diagnostics (CR), while the other 2 authors mainly conduct research in acute and intensive care (LB and FS). Finally, 4 authors have a doctorate in nursing (CR, LB, RP, and S Metzing), and 1 author is currently working on his doctorate in this study (FS).

### Ensuring the Quality of This Study

Compliance with quality criteria is central to research. On the basis of the primarily qualitative research design of this study, the relevant quality criteria must be identified. Nevertheless, “a single and specific set of quality criteria is neither feasible nor anticipated” [63] due to the diverse paradigms in qualitative research. Therefore, researchers must reflect on their own project and subject matter and determine suitable criteria along various aspects [63].

On the one hand, the rigor and quality of the study relate to the 3 central aspects of ontology, epistemology, and methodology [64]. In contrast, it also involves “the steps of designing, conducting, and reporting qualitative research in a step-wise approach” [65].

Therefore, in this study, we are guided by 4 criteria to enrich our own trustworthiness [63]: credibility, transferability, dependability, and confirmability. These can essentially be achieved through reflexivity, peer involvement, triangulation of methods and data, prolonged engagement, persistent observation, maximum variation, typical sampling, respondent validation, data collection until saturation, and thick description [63]. Information on all these criteria can be found throughout this study protocol. In addition, we will consider the quality criteria in the subsequent publications on the results of this study.

Therefore, we will adhere to established guidelines when reporting the results. As there are currently no specific reporting guidelines for the development of program theories, we will follow the “Standards for Reporting Qualitative Research” [66] due to the qualitative focus of this study.

### Ethical Considerations

This study follows the ethical principles of the Declaration of Helsinki [67]. In this regard, an ethics application was submitted to the ethics committee of Witten/Herdecke University to ensure that the study was legally and ethically sound. We received a positive ethics vote from the committee on April 28, 2025 (S-109/2025).

As third parties are involved in this research project, and participants’ and personal data are collected, a data privacy management process was carried out at the university conducting the project and approved in March 2025. The contents are publicly accessible to the participants via the Deggendorf Institute of Technology website. It was agreed with the data protection officers that only the personal data that are absolutely necessary for the research project will be collected.

The ethics application and data protection management stipulated that all HCPs must sign an informed consent before the study, confirming their voluntary participation. The agreement also assured them that, once the study had been completed, all personal information would be immediately pseudonymized by the researchers and anonymized for third parties. After completion of the study, but no later than December 2026, all personal information and data collected will be irrevocably deleted. During the study and the analysis, participants will have the opportunity to be informed about the processing of their data at any time. In addition, they can withdraw from the study until the results are published and retract their statements without giving reasons.

HCPs will not receive compensation for travel expenses or lost work time for their participation. However, catering will be provided on-site during the workshop days.

### Dissemination Policy

We plan to publish 2 open-access and peer-reviewed articles on the results. One article will contain the core results of the group discussions and workshops as a separate, empirical report. The second article will include the revised and finalized program theory developed in this study. We will also present the results at national and international scientific conferences. Finally, we intend to actively approach people in clinical care and disseminate the results to the ICUs there.

## Results

The initial program theory, which was developed in the first step of this study from March 2023 to May 2025, will be presented subsequently. This will serve as the basis for the empirical review, revision, and refinement conducted in the second step of this study.

### Defining the Core Problem

**Figure 2:**
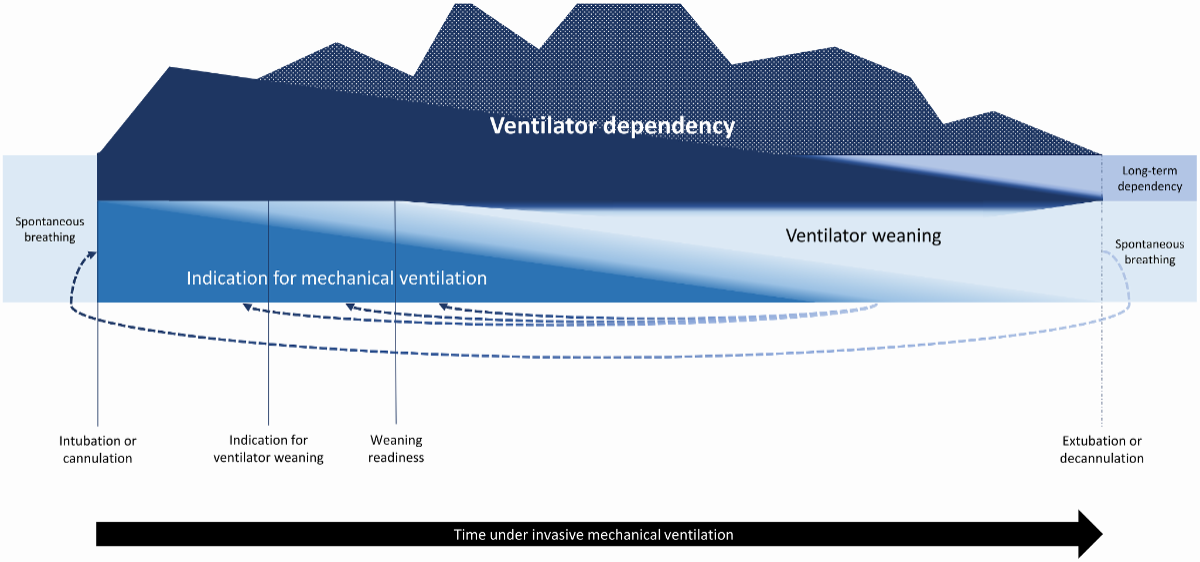
The phenomenon of ventilator dependency.

This program addresses ventilator dependency (Figure 2). While the starting point in this case is ongoing spontaneous breathing, reasons arise for an intubation or cannulation of patients. These causes are linked to a pronounced indication for MV. In this first period, patients are dependent on respiratory support to some degree, but the dependency increases along with the duration of MV, substance use, and the loss of muscles. Therefore, the core problem is not the ventilation itself, but the iatrogenic dependence on the ventilator.

While the indication for MV decreases, the importance of required efforts for ventilator weaning increase over time. Their transition is fluid, and the processes can run in parallel. Nevertheless, an indication for ventilator weaning must be given initially, and weaning readiness must then be achieved so that the weaning process can be initiated.

In the meantime, the ventilator dependency ideally decreases continuously but can also change in the course of time. During the weaning process, patients may also fall behind in their progress, no longer be ready for weaning, or have their indication for weaning withdrawn.

Finally, patients are ideally no longer dependent on the ventilator, are extubated, and can breathe spontaneously again. However, ventilator dependency can also manifest itself and turn into long-term dependency. Ultimately, patients can also experience weaning failure after extubation and undergo reintubation. At this point, the process starts all over again.

### Focusing and Scoping of the Program

The main interest of this program theory lies in facilitating ventilator liberation. Extubation or decannulation in conjunction with sufficient spontaneous breathing is its key goal. The setting of interest is the ICU in acute inpatient care. The main actors in this regard are HCPs who are responsible for the treatment and ventilator weaning of the affected patients (eg, intensivists, critical care nurses, and therapists). In addition, not only the patients themselves and their relatives and friends but also pastoral workers, cleaning staff, and hospital management play an important role.

### Key Outcomes in Ventilator Weaning

**Figure 3:**
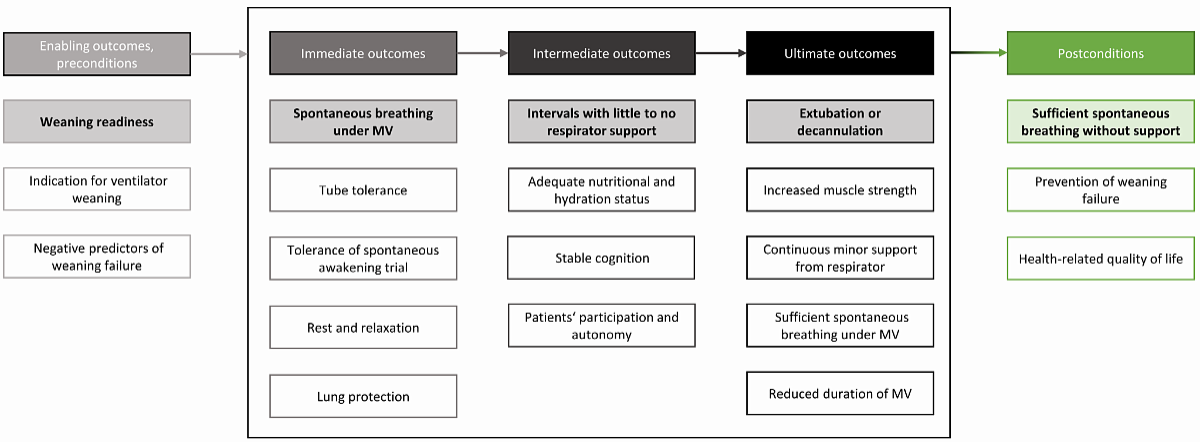
Key outcomes of ventilator weaning. MV: mechanical ventilation.

Before the initiation of the program, preconditions need to be fulfilled (Figure 3). Patients need to be ready for a ventilator weaning and have no signs of possible weaning failure. In addition, there must be an indication for ventilator weaning.

During ventilator weaning, immediate, intermediate, and ultimate outcomes manifest themselves consecutively. These should not only be achieved at the respective point in time but should also be continuously maintained from then on. The outcomes build on each other and are a prerequisite for the next level. For instance, patients must be able to breathe spontaneously under MV in the short term so that the duration of spontaneous breathing can become longer in the medium term, and they can be extubated in the long term.

After extubation and completion of the program, postconditions that are needed to maintain the patient’s stable situation become relevant. This includes sufficient spontaneous breathing without support, the prevention of weaning failure, and a preserved health-related quality of life.

### Key Interventions in Ventilator Weaning

**Figure 4:**
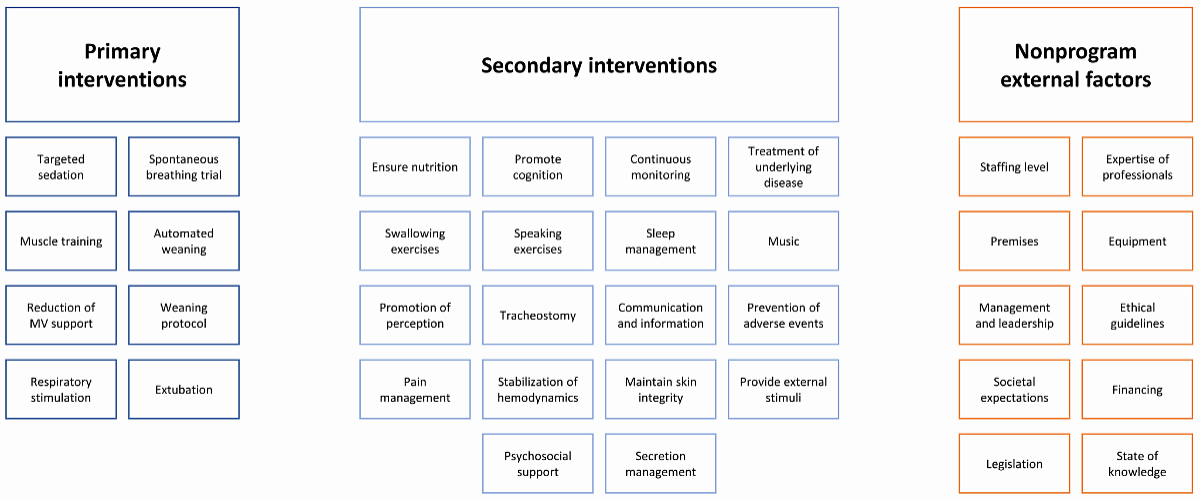
Key interventions of ventilator weaning. MV: mechanical ventilation.

This program incorporates primary interventions—such as spontaneous breathing trial, muscle training, or the reduction of MV support—that have a direct impact on the outcomes (Figure 4). The secondary interventions have an indirect impact on the outcomes. For instance, nutrition, promotion of perception, or pain management enable spontaneous breathing trials or muscle training and thus have an influence on the key outcomes but do not have a direct effect on the latter.

In addition to these interventions, nonprogram external factors also affect the outcomes but are beyond the control of the program managers. In addition to the staffing level or the equipment on the premises, these also include legislation and the funding system.

### Interaction of Program and Outcomes

**Figure 5:**
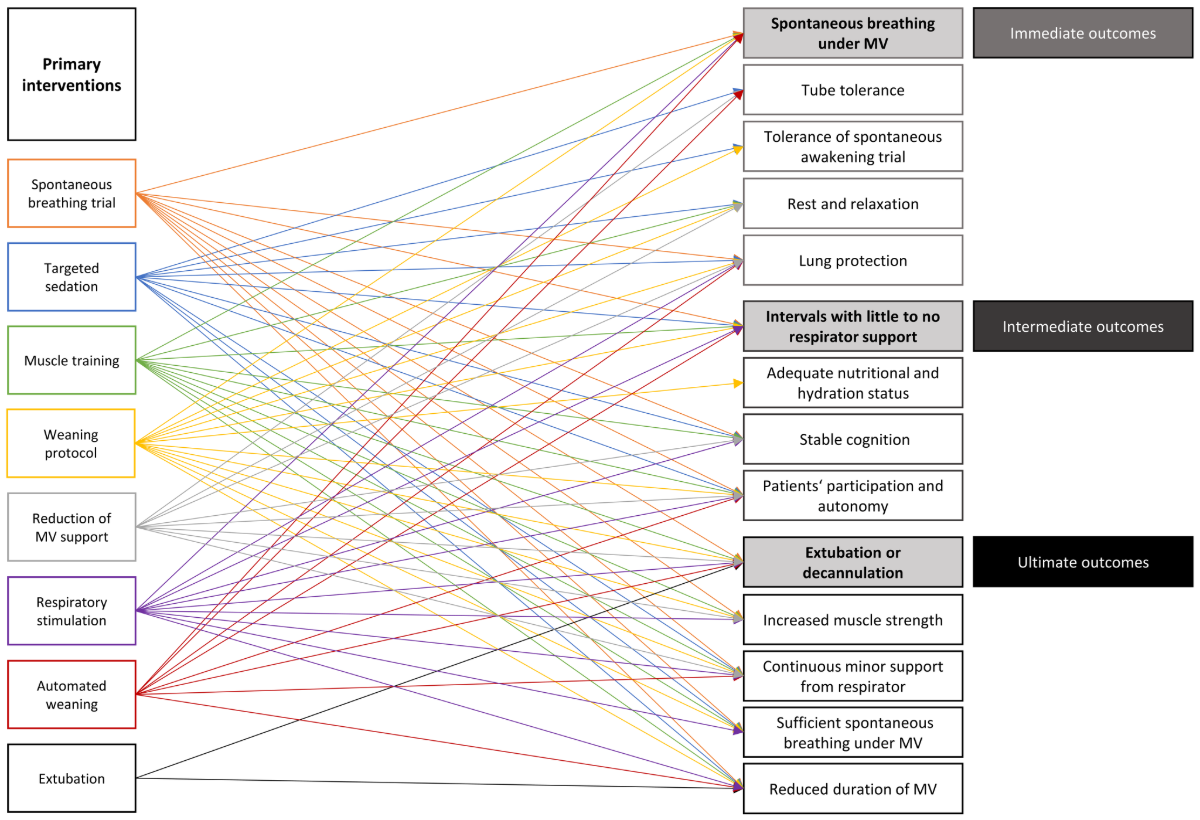
Linking primary interventions and outcomes. MV: mechanical ventilation.

The complexity of ventilator weaning in adult intensive care patients unfolds in the compilation of the central outcomes and interventions (Figure 5). Individual interventions influence different outcomes at different points in time, which, in turn, are prerequisites for each other. However, not every outcome is targeted equally, and not every intervention has an impact on several endpoints.

### Expected Results From Step 2

In the second step of this study, the aforementioned initial program theory will be further developed and refined. The revised program theory will provide a complex theoretical understanding of ventilator dependency as the core problem and ventilator weaning as an appropriate response. To this end, the ToA and the ToC will be advanced and combined in a logic model. The necessary data are being collected in an iterative process and will be evaluated in 2026. The results and the final program theory are to be presented in 2 publications planned for the same year.

## Discussion

### Anticipated Findings

The aim of this study is to develop a program theory on ventilator weaning in adult intensive care patients. To this end, we have designed a comprehensive research program that is methodologically based on 2 steps and follows the current recommendations of the MRC framework [32,46]. The initial program theory presented in this paper serves as the basis for the empirical review and already differentiates between the core phenomenon, a variety of outcomes, multiple interventions, and the interconnection of these elements.

This procedure has not yet been described in this way and is unique in its combination of methods. This is also based on the fact that the development of program theories is still rare internationally. In addition, the methodological guidelines are often kept at an abstract and general level. Therefore, it is important to make methodological approaches transparent and to propose these procedures to the scientific community.

It can be assumed that the development and publication of this theory will lead to a differentiated understanding of ventilator weaning and sustainable research in science and clinical care. As mentioned by the MRC framework, program theories are not definitive and permanent. They should be further developed in the course of intervention development and testing as well as adapted to changes in health care over the years. In this regard, our revised program theory must undergo a critical examination in clinical practice. The next steps, along with the MRC framework, are piloting, evaluation, and implementation. At each stage, the program theory should be reviewed and refined as necessary [32]. In this way, program theory enables the development and manifestation of ventilator weaning as a complex intervention. We will pursue this aim in a subsequent research project.

### Strengths

Several experts in critical care and social sciences are involved in the development of this theory, which is a key strength of the study. The careful consideration of the literature in step 1 provides the theory with a sound basis and makes it relevant to clinical practice and existing research. The iterative process in step 2 enables not only a one-off but repeated confirmation and refinement of the core ideas of this theory development. Moreover, all steps in the development of the theory were reported transparently and sequentially. This not only enables traceability but also further development of the method and theory.

### Limitations

This study also has limitations. First, participants are recruited exclusively from German-speaking countries. HCPs from other countries are not included in this study. Second, no quantitative methods are used to review the theory at this stage. This is due to the fact that Funnell and Rogers [46] primarily recommend stakeholder discussions and workshops. In addition, a broad body of literature with various statistical evaluations was incorporated into the theory development in the initial phase. Third, ICU patients receiving MV are diverse. They differ in terms of their primary diagnosis and comorbidities, age, and course of the disease as well as central treatment recommendations. What they all have in common is ventilator dependency as the central phenomenon. However, the extent to which the program theory can actually consider the heterogeneity of the patients or whether subgroups need to be formed will become clear in the course of the study. Fourth, no patients or relatives are to be included in the revision of this theory. This may reinforce professional perspectives in the theory and leave aspects unaddressed. However, this step was taken deliberately. As these patients constitute a vulnerable group [68], it should be carefully evaluated whether they should be studied (again) at all [69]. In our meta-synthesis [54], we found that there are already a large number of studies on the experience of MV and ventilator weaning whose data we already have access to. In addition, it turned out that the patients could not consciously distinguish between the processes of MV and ventilator weaning [27,70]. This could result in more bias in the study than potential benefit.

### Conclusions

The initial program theory confirms ventilator weaning as a complex intervention and a multidisciplinary field in which nurses play a central role. After its revision, it can lead to a differentiated understanding that supports the HCPs in the ICUs in their daily work and creates sensitivity for relevant outcomes and appropriate interventions. These can be specifically combined, coordinated, and better evaluated. On the basis of this program theory, ventilator weaning can be developed, piloted, evaluated, and implemented as a complex intervention in the sense of the MRC framework.

## Appendix

Multimedia Appendix 1 Interview guides for stakeholder conversations (step 1) as well as group discussions and workshops (step 2).

## Abbreviations

HCP: health care professional
ICU: intensive care unit
MRC: Medical Research Council
MV: mechanical ventilation
SPIRIT: Standard Protocol Items: Recommendations for Interventional Trials
ToA: theory of action
ToC: theory of change
WIND: Weaning According to a New Definition
WEAN SAFE: Worldwide Assessment of Separation of Patients From Ventilatory Assistance

## Data Availability

The datasets generated or analyzed during this study are available from the corresponding author on reasonable request.
