# Supplementary material for: Developing a Program Theory on Ventilator Weaning in Adult Intensive Care: Protocol for a Multimethods Study
Source: JMIR Res Protoc. 2026 Jan 30;15:e83342. doi: 10.2196/83342 (PMC12905570; doi:10.2196/83342)
Supplement: Multimedia Appendix 1 [file resprot_v15i1e83342_app1.pdf]

Supplement 1: Interview guides

All interview and workshop guidelines are originally in German. The translations here serve the transparency of the research process.

Guide 1 – Stakeholder conversation (initial program theory)

All subsequent impulse questions served to explicate the mental models and understanding of mechanical ventilation and ventilator weaning of the various stakeholders. The order and wording of the questions could vary.

<u>Running order (semi-structured, questions can be asked flexibly)</u>

- What causes respiratory insufficiency?
- What is the indication for mechanical ventilation?
- What is problematic about mechanical ventilation?
- What consequences can result from mechanical ventilation?
- For whom does the problem exist? Who is affected?
- What is weaning failure? How is it defined?
- How does weaning failure occur?
- What are the short-, medium- and long-term endpoints that are relevant to weaning?
- What exactly do these endpoints look like? What attributes do they have?
- What starting point must be present in order to take action? (Indication for weaning?)
- Which measures are part of weaning?
- Which measures also influence weaning but cannot be influenced by healthcare professionals?
- Who is involved in weaning?
- Who is responsible for which tasks and areas?

Guide 2 – Group discussion (revising program theory)

Running order (semi-structured, questions can be asked flexibly)

Topic 1 - Preconditions

- What is required for ventilator weaning to be initiated?

Topic 2 - Outcomes

- What are the main goals of ventilator weaning? What is it aimed at?
- How can these goals be described? What are their characteristics?
- At what point in time are the goals relevant?
- Should the goals be achieved once or continuously?

Topic 3 - Interventions

- What are key interventions for ventilator weaning?
- How should they be carried out?
- When should they be carried out?

Topic 4 - Context

- What goals do these interventions affect?
- What external factors influence ventilator weaning?

Guide 3 – Workshop on outcomes (revising program theory)

The participants are given the individual outcomes from the initial program theory printed out on A4 paper and are asked to assign them to the main categories (precondition, immediate, intermediate, ultimate, postcondition). They are allowed to critically assess both the categorization and the outcomes. They should justify their decision, remove unsuitable outcomes and add missing outcomes on blank pieces of paper.

Guiding questions during the workshop are:

- Do you think the goals presented here are relevant?
- Are you missing a goal?
- Would you like to remove one of the goals?
- How should these goals be achieved?
- When should they be achieved?
- Are they in the right order and direction?

Guide 4 – Workshop on interventions (revising program theory)

The participants are given the individual interventions from the initial program theory printed out on A4 paper and are asked to assign them to the main categories (primary, secondary, non-program external). They are allowed to critically assess both the categorization and the interventions. They should justify their decision, remove unsuitable interventions and add missing interventions on blank pieces of paper.

Guiding questions during the workshop are:

- Do you think the interventions presented here are relevant?
- Are you missing an intervention?
- Would you like to remove one of the interventions?
- How should these interventions be carried out?
- When should these interventions be carried out?
- Are these interventions sorted correctly?
- Which goals are influenced by which intervention?
